# Evaluation of plasma levels of NFL, GFAP, UCHL1 and tau as Parkinson's disease biomarkers using multiplexed single molecule counting

**DOI:** 10.1038/s41598-023-32480-0

**Published:** 2023-03-30

**Authors:** Priscilla Youssef, Laura Hughes, Woojin S. Kim, Glenda M. Halliday, Simon J. G. Lewis, Antony Cooper, Nicolas Dzamko

**Affiliations:** 1 Faculty of Medicine and Health and the Brain and Mind Centre, School of Medical Sciences, University of Sydney, Camperdown, NSW 2050 Australia; 2 Garvan Institute of Medical Research, Darlinghurst, NSW 2010 Australia; 3 St Vincent’s Clinical School, UNSW-Sydney, Darlinghurst, NSW 2010 Australia

**Keywords:** Neuroscience, Diseases of the nervous system, Parkinson's disease

## Abstract

Objective biomarkers for Parkinson’s Disease (PD) could aid early and specific diagnosis, effective monitoring of disease progression, and improved design and interpretation of clinical trials. Although alpha-synuclein remains a biomarker candidate of interest, the multifactorial and heterogenous nature of PD highlights the need for a PD biomarker panel. Ideal biomarker candidates include markers that are detectable in easily accessible samples, (ideally blood) and that reflect the underlying pathological process of PD. In the present study, we explored the diagnostic and prognostic PD biomarker potential of the SIMOA neurology 4-plex-A biomarker panel, which included neurofilament light (NFL), glial fibrillary acid protein (GFAP), tau and ubiquitin C-terminal hydrolase L1 (UCHL-1). We initially performed a serum vs plasma comparative study to determine the most suitable blood-based matrix for the measurement of these proteins in a multiplexed assay. The levels of NFL and GFAP in plasma and serum were highly correlated (Spearman rho-0.923, *p *< 0.0001 and rho = 0.825, *p *< 0.001 respectively). In contrast, the levels of tau were significantly higher in plasma compared to serum samples (*p *< 0.0001) with no correlation between sample type (Spearman *p *> 0.05). The neurology 4-plex-A panel, along with plasma alpha-synuclein was then assessed in a cross-sectional cohort of 29 PD patients and 30 controls. Plasma NFL levels positively correlated with both GFAP and alpha-synuclein levels (rho = 0.721, *p *< 0.0001 and rho = 0.390, *p *< 0.05 respectively). As diagnostic biomarkers, the control and PD groups did not differ in their mean NFL, GFAP, tau or UCHL-1 plasma levels (t test *p *> 0.05). As disease state biomarkers, motor severity (MDS-UPDRS III) correlated with increased NFL (rho = 0.646, *p *< 0.0001), GFAP (rho = 0.450, *p *< 0.05) and alpha-synuclein levels (rho = 0.406, *p *< 0.05), while motor stage (Hoehn and Yahr) correlated with increased NFL (rho = 0.455, *p *< 0.05) and GFAP (rho = 0.549, *p *< 0.01) but not alpha-synuclein levels (*p *> 0.05). In conclusion, plasma was determined to be most suitable blood-based matrix for multiplexing the neurology 4-plex-A panel. Given their correlation with motor features of PD, NFL and GFAP appear to be promising disease state biomarker candidates and further longitudinal validation of these two proteins as blood-based biomarkers for PD progression is warranted.

## Introduction

Parkinson’s disease (PD) is a progressive neurodegenerative disorder resulting from the loss of dopaminergic neurons in the substantia nigra in association with the accumulation of alpha-synuclein-rich protein aggregates in multiple brain regions. Clinically, PD presents with non-motor symptoms such as gastrointestinal dysfunction, hyposmia and sleep disturbances that can present years before the onset of the classical PD motor symptoms of bradykinesia, rigidity and tremor^1^. The diagnosis of PD currently relies on the presentation of these heterogenous clinical symptoms, which makes early and accurate diagnosis challenging^2^, and highlights the need for the development and validation of objective biomarkers for both PD diagnosis and monitoring of PD progression.

Neurofilament light (NFL) is one such protein that has gained significant interest as a potential blood-based biomarker for PD using the single molecule array (SIMOA) platform. Neurofilaments are structural proteins that comprise a significant portion of neuronal axons, and can leak into extracellular compartments, including blood, upon axonal injury^3^. Studies have now demonstrated that higher serum NFL levels are a marker of neurodegeneration, and can discriminate PD from controls, discriminate PD from other synucleinopathies, and even between PD patients with and without cognitive impairment^4–6^.

NFL can be multiplexed in a SIMOA assay with other markers of brain degeneration, namely glial fibrillary acid protein (GFAP), ubiquitin C-terminal hydrolase L1 (UCHL1) and total tau. GFAP is a major structural component of fibrillary astrocytes that are induced during astrogliosis following CNS injuries and neurodegeneration. GFAP is rapidly released into biofluids, including CSF and peripheral blood, and has also emerged as a candidate biomarker for neurodegenerative disorders^7^. While its measurement using the SIMOA platform has been well documented in the context of dementia, showing an increase in both Alzheimer’s disease (AD)^8^ and frontotemporal dementia (FTD) patients^9,10^, along with patients with pre-symptomatic stages of AD^11^, studies of GFAP in the context of PD have been limited. UCHL1 is a deubiquitinating enzyme predominantly expressed in neurons that plays an important role in the degradation of abnormally deposited proteins. UCHL1 has been associated with higher MDS-UPDRS III motor scores suggesting a potential role as a marker of PD progression^12^. Additionally, plasma levels of tau (a microtubule-associated protein localized primarily in neurons and traditionally associated with AD^13^), have been suggested to increase the diagnostic accuracy of PD, with elevated tau levels thought to predict cognitive impairment in PD patients prior to symptomatic onset^14,15^.

Whilst these degenerative associated proteins have each displayed potential as PD biomarkers, they have been predominantly assessed individually across different cohorts, and largely in CSF. Therefore, the present study aimed to determine the diagnostic and prognostic potential of the neurology 4-plex-A assay markers NFL, GFAP, UCHL1 and tau in blood samples of a single cohort of PD patients and healthy controls using the ultra-sensitive SIMOA technology.

Whilst previous studies suggest that NFL and GFAP levels are relatively stable and translatable between serum and plasma samples^16–18^ , total tau levels have been reported to be more stable in plasma samples^19^, whilst UCHL1 measures between serum and plasma remain largely undetermined. As pre-analytical variation and sample selection is known to influence biomarker levels, we therefore first performed a serum vs plasma comparative study to determine the most suitable blood sample type for the neurology 4-plex-A biomarkers. Levels of NFL, GFAP, UCHL1 and tau were then assessed in a cross-sectional cohort of PD patients and matched controls to determine biomarker utility.

## Results

### Assessment of GFAP, NFL, UCHL1 and tau levels in plasma versus serum

**Figure 1:**
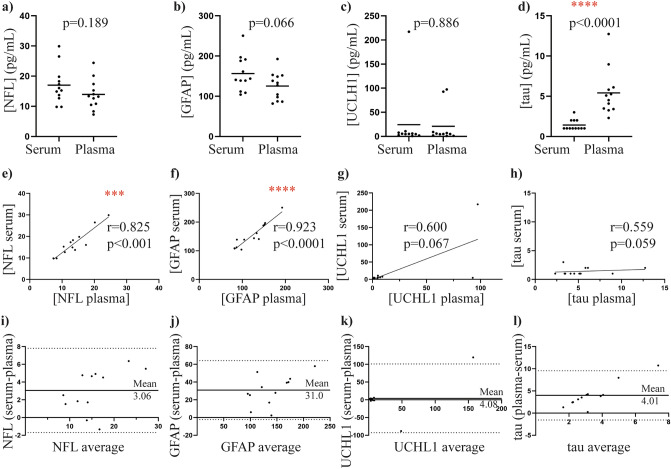
Biomarker levels in serum vs plasma samples. Biomarker levels in serum and plasma samples of 12 participants are displayed. Individual values plots display non-parametric t-test (Mann Whitney U) results, revealing no significant differences between serum and plasma levels of (**a**) NFL, (**b**) GFAP and (**c**) UCHL1, and a significant difference between (**d**) serum and plasma tau levels. Scatter plots were used to display Spearman’s correlations between serum and plasma matrices, with significance at the 0.05 level. A significant positive correlation was found between (**e**) serum and plasma NFL and (**f**) serum and plasma GFAP. No significant correlations were observed between (**g**) serum and plasma UCHL1 levels (n = 11) and (**h**) serum and plasma tau levels. Bland Altman plots further display the relationship between serum and plasma levels of (**i**) NFL, (**j**) GFAP, (k) UCHL1 (n = 11) and (l) tau, with plots displaying the mean and upper and lower limits of the 95% confidence interval. ***p < 0.01, ****p < 0.0001.

All measured biomarkers were within the detectable range in all samples and when assay performance was assessed, NFL and GFAP intra-assay CV% were < 12% for all samples (Table 1). Of the total tau samples, 7/12 serum samples had a CV% < 12% and 9/12 plasma samples had a CV% < 12%. For UCHL1 samples, 1/6 serum samples had a CV% < 12% and 5/7 plasma samples with a CV% < 12% (Table 1). There were no significant differences between the plasma and serum levels for NFL, GFAP and UCHL1 (all *p *> 0.05, Fig. 1A-C respectively), although serum NFL and GFAP levels were slightly higher than their plasma levels. In contrast, significantly less total tau was observed in serum (1.4 ± 0.2) compared to plasma samples (2.9 ± 0.8) (*p *< 0.0001, Fig. 1D). Spearman correlations revealed positive correlations between serum and plasma NFL levels (rho = 0.825, *p *< 0.001, Fig. 1E) and between serum and plasma GFAP levels (rho = 0.923, *p *< 0.0001, Fig. 1F). No correlations were observed between serum and plasma UCHL1 levels or serum and plasma tau levels (*p *> 0.05, Fig. 1G–H respectively). Bland–Altman plots were also performed to assess the general agreement between the same biomarkers measured in plasma and serum (Fig. 1I–L). These plots indicate the level of bias when one biofluid is used over the other and suggest that results between the two biofluids may not be readily interchangeable.

**Table 1:** Serum and plasma intra-assay precision.

	NFL	GFAP	tau	UCHL1
Average CV% (range) (serum)	3.1 (0.1—11.6)	1.8 (0.3—5.9)	14.0 (5.1—45.8)	53.2 (11.9—130.9)
CV% < 12% /total samples (serum)	12/12	12/12	7/12	1/6
Average CV% (range) (plasma)	3.8 (0.04—10.9)	2.0 (0.4—3.6)	8.9 (0.7—27.7)	9.4 (0.1—26.1)
CV% < 12% /total samples (plasma)	11/11	11/11	8/11	6/8

The mean concentrations and range of measured biomarker analytes were measured in serum and plasma. High intra-assay precision was reflected by a CV < 12%. Total sample numbers less than 12 reflect cases where samples duplicates could not be obtained.

### Assessment of plasma GFAP, NFL, tau, UCHL1 and alpha-synuclein in controls versus PD

**Figure 2:**
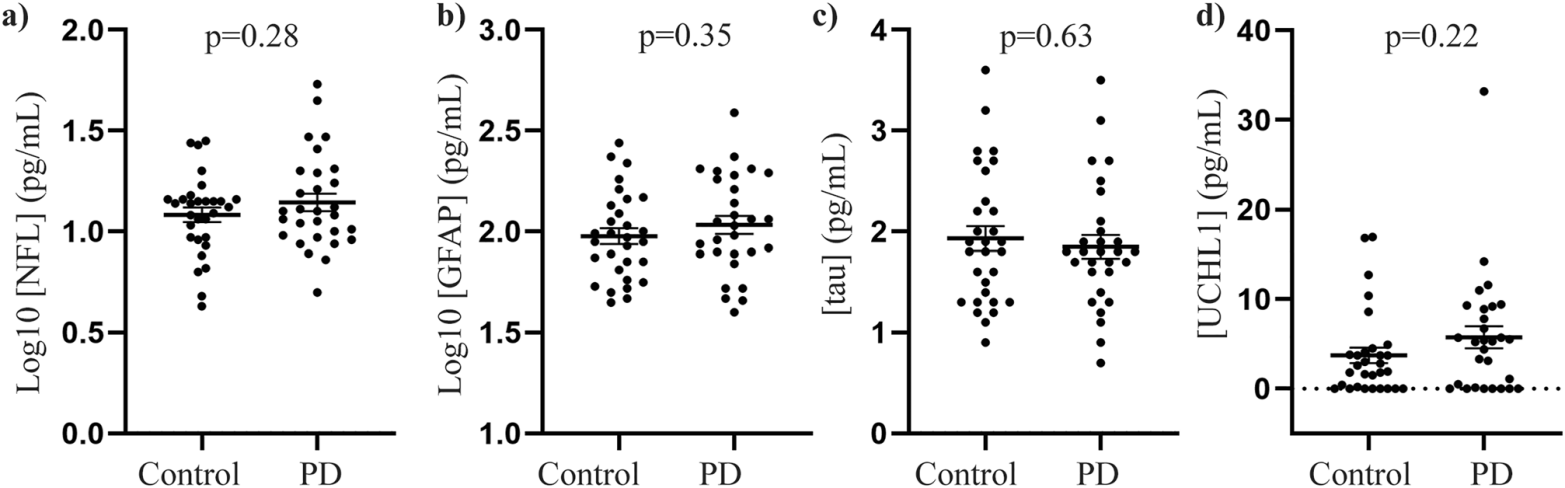
Assessment of NFL, GFAP, tau and UCHL1 in plasma samples of PD patients and controls. Individual value plots display biomarker concentrations in plasma samples of the control (n = 30) and PD (n = 29) groups. Where indicated, concentration values were log transformed to achieve normality, and a Parametric t-test was used to assess differences in biomarker levels between groups. Parametric t-tests revealed no significant differences between plasma levels of (**a**) NFL, (**b**) GFAP and (**c**) tau levels. Further, a non-parametric t-test (Mann Whitney U) revealed no significant difference in levels of (**d**) UCHL1 in the PD group relative to the control. Graphs also display the mean ± SEM.

The PD patients and controls enrolled in the study were matched for age and sex (Table 2). There were no significant differences between the concentrations of NFL, GFAP, total tau or UCHL1 in the PD group compared to controls (Fig. 2A–D respectively). A multivariate analysis covarying for age and sex was also employed to determine whether these variables were influencing the results. However, again no significant differences between concentrations of the GFAP, NFL and tau in plasma samples of the PD group relative to the control group were found (Supplementary Figure S1A–C respectively). UCHL1 could not be transformed to achieve a normal distribution and was not included in the multivariate analysis. There was also no significant difference in either total alpha-synuclein or Hgb levels between the groups (Table 2). Additionally, total alpha-synuclein levels were covaried for age, sex and Hgb levels as previously suggested^21^, with no differences found between PD and control groups (Table 2). We also examined if ratios of GFAP:alpha-synuclein, NFL:alpha-synuclein or NFL:GFAP could enhance biomarker potential, but still no significant difference was observed between control and PD groups (Supplementary Figure S1D–E).

**Table 2:** Demographic and clinical details.

	Control (n = 30)	PD (n = 29)	*p*-value
Age (years)	68 ± 1.8 (45–87)	68 ± 1.7 (42–83)	0.92
Sex (% M)	67%	72%	0.64
Years since diagnosis	N/A	6.9 ± 0.8 (0–12)	
H&Y stage	N/A	2 ± 0.1 (1–4)	
MDS-UPDRS III	N/A	27 ± 3.0 (5–76)	
Total alpha-synuclein	12.1 ± 0.9	14.0 ± 1.7	0.32
Hgb (µg/ml)	53.7 ± 7.7	47.4 ± 7.6	0.55
Log10_Total alpha-synuclein (covarying for age, sex and Hgb levels)	1.037 ± 0.034	1.068 ± 0.044	0.689

Demographic and clinical details of the participants who donated the plasma samples used in this study. Data are mean ± standard error, with the range shown in parentheses. Disease severity was recorded using the Hoehn and Yahr (H&Y) scale

*MSD-UPDRS* Movement disorder society-unified Parkinson's disease rating scale. *Hgb* Hemoglobin. *NA* Not applicable.

### Correlations between plasma GFAP, NFL, tau, UCHL1 and alpha-synuclein

**Figure 3:**
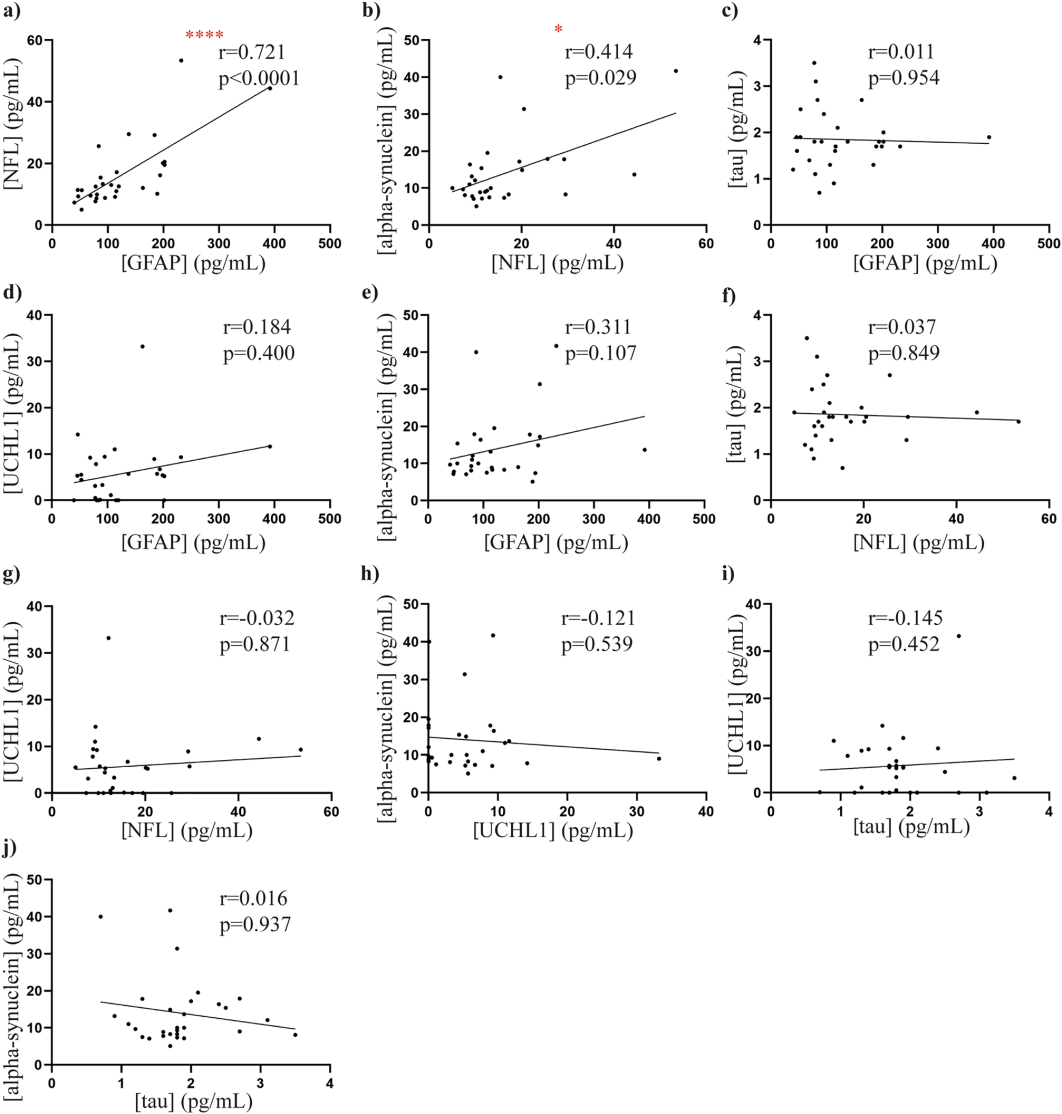
Correlations between plasma biomarkers in PD patients. Scatter plots display correlations between plasma biomarkers in the PD group (n = 29). Spearman’s correlations were used with significance at the 0.05 level. A significant positive correlation was found between (**a**) NFL and GFAP and (**b**) NFL and alpha-synuclein. No significant correlations were observed between (**c**) GFAP and Tau (**d**) GFAP and UCHL1, (**e**) GFAP and alpha-synuclein, (**f**) NFL and Tau, (**g**) NFL and UCHL1, (**h**) alpha-synuclein and UCHL1, (**i**) Tau and UCHL1, and (**j**) tau and alpha-synuclein. *p < 0.05, ****p < 0.001.

There was a significant positive correlation between NFL and GFAP levels (rho = 0.731, *p *< 0.0001, Fig. 3A) and between NFL and total alpha-synuclein levels (rho = 0.414, *p *< 0.05, Fig. 3B). No further significant correlations were observed between the levels of GFAP, NFL, tau, UCHL1 or alpha-synuclein (all *p *> 0.05, Fig. 3C–J).

### Correlations between plasma biomarkers and clinical variables

**Figure 4:**
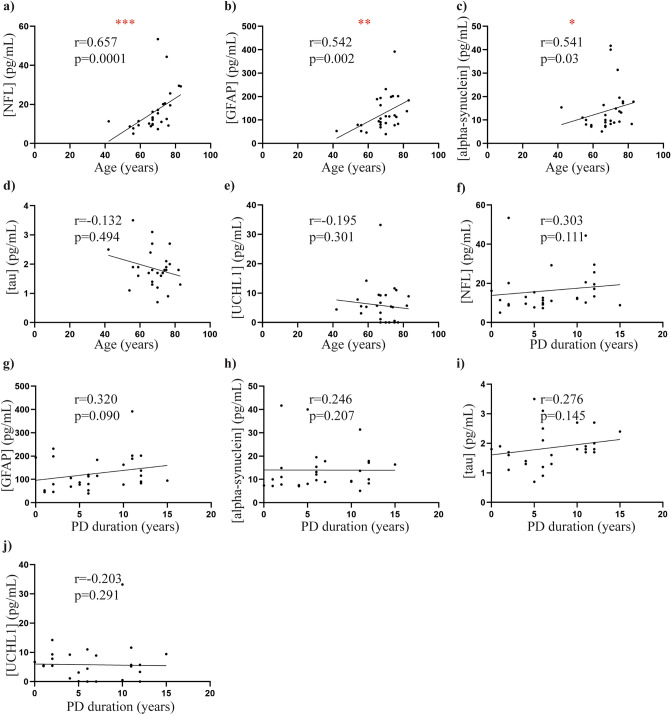
Correlations between plasma biomarkers and demographic data. Scatter plots show correlations between plasma biomarkers and demographic data in the PD cohort (n = 29). Spearman’s correlations were used with significance at the 0.05 level. A significant positive correlation was found between (**a**) age and NFL (**b**) age and GFAP, and (**c**) age and alpha-synuclein. No significant correlations were observed between age and (**d**) tau and (**e**) UCHL1. Further, no significant correlations were observed between disease duration and (**f**) NFL, (**g**) GFAP, (**h**) alpha-synuclein, (**i**) tau and (**j**) UCHL1. *p < 0.05, **p < 0.01, ***p < 0.001.

**Figure 5:**
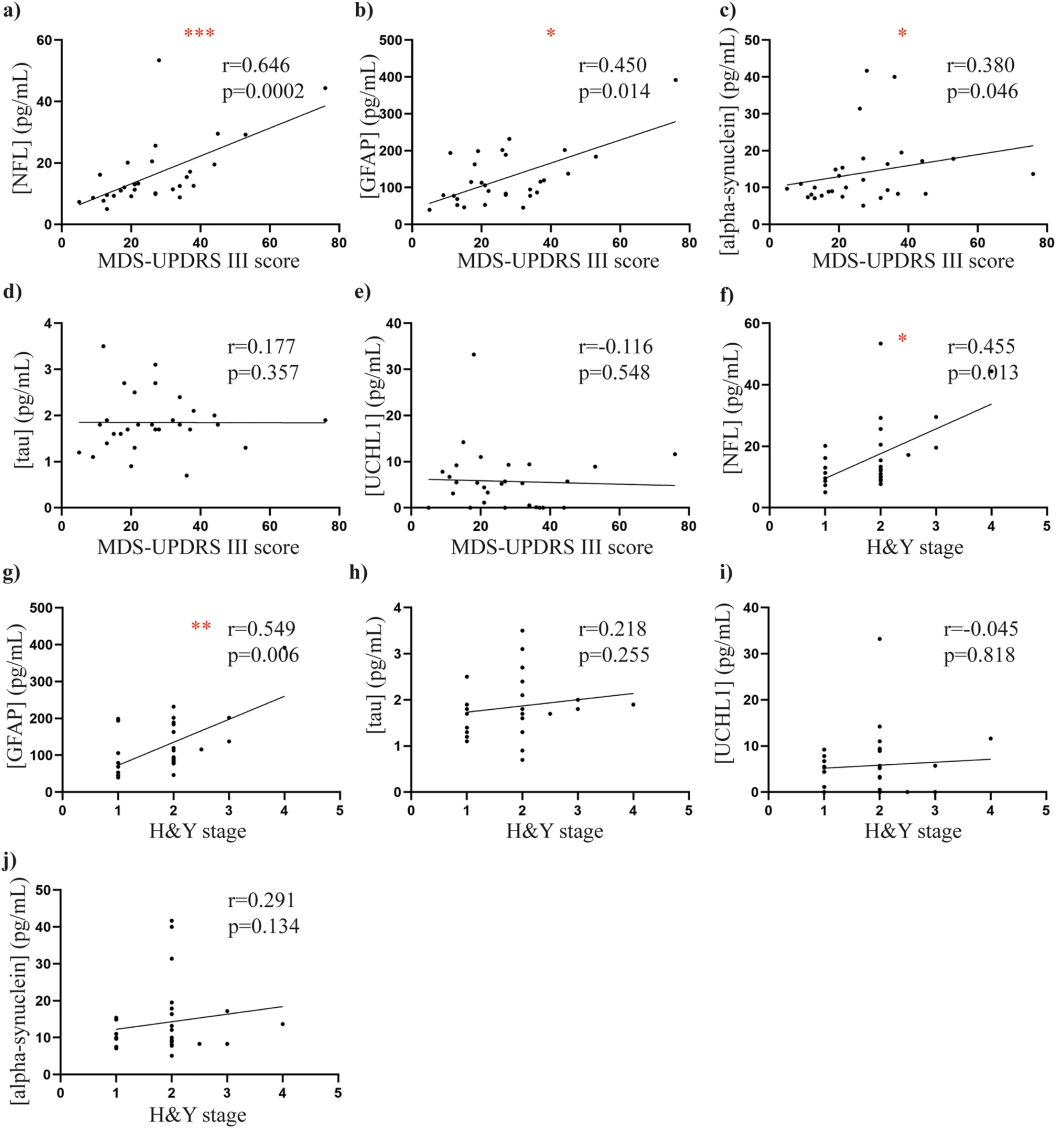
Correlations between plasma biomarkers and motor severity scales. Scatter plots show correlations between plasma biomarkers and motor severity scales in the PD cohort (n = 29). Spearman’s correlations were used with significance at the 0.05 level. A significant positive correlation was found between (**a**) MDS-UPDRS III score and NFL, (**b**) MDS-UPDRS III score and GFAP, and (**c**) MDS-UPDRS III score and alpha-synuclein. No significant correlations were observed between MDS-UPDRS III score and (**d**) tau and (**e**) UCHL1. Further, a significant correlation was found between (**f**) H&Y stage and NFL and (**g**) H&Y and GFAP. No significant correlations were observed between H&Y stage and (**h**) tau, (**i**) UCHL1 and (**j**) alpha-synuclein. *p < 0.05, **p < 0.01, ***p < 0.001.

In the PD group, there was a significant positive correlation between age and plasma NFL (rho = 0.6569, *p *< 0.0001, Fig. 4A, plasma GFAP (rho = 0.5421, *p *< 0.01, Fig. 4B), and plasma alpha-synuclein (rho = 0.541, *p *< 0.05, Fig. 4C). No other significant correlations were observed between the plasma biomarkers and demographic data in the PD group (all *p *> 0.05, Fig. 4D-J). The severity of motor symptoms measured by the MDS-UPDRS III score positively correlated with NFL (rho = 0.646, *p *< 0.001, Fig. 5A), GFAP (rho = 0.450, *p *< 0.05, Fig. 5B) and total alpha-synuclein levels (rho = 0.380, *p *< 0.05, Fig. 5C), but not with levels of tau or UCHL1 (both *p *> 0.05, Fig. 5D-E). Increasing disease stage measured by the H&Y stage also positively correlated with NFL (rho = 0.455, *p *< 0.05, Fig. 5F) and GFAP levels (rho = 0.5496, *p *< 0.01, Fig. 5G). No further significant correlations were observed between plasma biomarkers and motor severity or stage (all *p *> 0.05, Fig. 5H-J).

## Discussion

Objective biomarkers for PD are considered critical for enabling earlier diagnosis, effective monitoring of disease progression, improved clinical trial design and interpretation of novel PD treatment efficacy. Ideal biomarkers include those that are easily accessible and detectable in patients, and that reflect the underlying pathology of PD; yet no such validated biomarkers are available to date. Whilst total alpha-synuclein remains a candidate biomarker of interest, previous reports have shown small overall differences and significant overlap in PD and control alpha-synuclein levels^22–24^. Moreover, it has been suggested that total alpha-synuclein measurements may better serve to standardise pathological forms of alpha-synuclein measurements in blood^25^ and/or serve as a marker of PD progression^26^. Notably, the presence of alpha-synuclein in atypical parkinsonism, alongside the heterogenous nature of PD symptoms and progression, suggests that a panel of biomarkers are required in the PD field. In the present study, we therefore evaluated the diagnostic and disease state biomarker potential of NFL, GFAP, total-tau and UCHL1 in a PD and age and sex matched control cohort using the ultra-sensitive SIMOA platform.

To determine whether plasma or serum samples should be used for the biomarker study, we first performed a serum vs plasma comparative study. The study showed no significant difference between serum and plasma NFL and GFAP levels. Additionally, both serum and plasma NFL and GFAP levels were significantly correlated, and although serum NFL and GFAP levels were slightly higher across all samples (approximately 1.3-fold increase), both serum and plasma NFL and GFAP levels were within detectable range and demonstrated overall excellent intra-assay precision (< 12%). Together our results suggest that NFL and GFAP are suitable for measurement in either serum or plasma samples. Our results align with previous studies which show that NFL serum and plasma levels are highly correlated^16,17^, and that serum NFL levels are slightly higher than in plasma^27–29^. Although previous studies have reported GFAP serum and plasma levels to be highly correlated^18^ and lower in EDTA plasma compared to serum^29^, our study is the first to demonstrate this in a simultaneous run, and more specifically, in a cohort that included both control and PD patients. Previous reports suggest that differences in biomarker levels between serum and plasma could be influenced by patient disease status (or lack thereof)^18,29^, further suggesting the need for the inclusion of both controls and movement disorder patients used in the present study.

In contrast, total-tau levels were significantly higher in plasma vs serum samples, with no overall correlation between plasma and serum total tau levels. Further, fold changes varied significantly across the cohort (ranging from a 1.1 to 9.0-fold increases in total tau plasma levels compared to serum), suggesting that plasma and serum samples are non-comparable and should be used independently. Further, total tau intra-assay analysis revealed higher variation among serum samples (7/12 samples with an intra-assay CV% < 12%, compared to 8/11 samples with an intra-assay CV% < 12% in corresponding plasma samples), suggesting that plasma should be used over serum where possible. This is in line with previous reports that have reported lower levels of total tau in serum vs plasma samples^19,30^, and that plasma total-tau levels are more stable than corresponding serum levels^19^. Thus, overall, our comparative study suggested plasma to be the optimal choice for the neurology 4-plex-A biomarker panel.

When diagnostic potential was assessed, none of the individual plasma biomarkers were effective at distinguishing PD from controls, even after covarying for age and sex. The data showed considerable overlap between patients and controls, as seen with plasma levels of alpha-synuclein both in our present study and previous reports^21,22^. Although there are limiting studies assessing GFAP and UCHL1 biomarkers potential in PD, our results are consistent with recent findings that serum^15^ or CSF^31^ GFAP levels are not significantly changed between PD and controls, although CSF GFAP levels were increased in one study^32^. A single study has also reported unchanged UCHL-1 plasma levels in PD and controls during early PD (H&Y < 2)^12^ whereas, to our knowledge, this is the first study to report on plasma total-tau levels in early staged PD patients using SIMOA. Interestingly, other studies have reported higher group levels of both CSF and serum NFL levels in PD compared to control cohorts, with this difference most likely attributable to increased sample size in these cohorts compared to our study (most studies report PD cohort sizes ranging from 70 to 397 patients)^4–6,33,34^. This suggests that only a proportion of patients may have higher NFL levels. Such patient heterogeneity in NFL levels has been highlighted in previous studies. For example, it has been shown that patients with postural instability and gait disorder (PIGD) have higher plasma NFL levels than patients with tremor dominant (TD) motor symptoms two years after baseline^6^. Also, PD patients with cognitive impairment have higher plasma NFL levels compared to cognitively normal patients^33,35^. Importantly, however, PD and control group differences are small, with high overlap of NFL levels across groups with most studies reporting fold changes between 1.08 and 1.84 in PD Nfl levels in CSF, serum, and plasma^4–6,34^. It has been suggested that the diagnostic accuracy of NFL may be improved when combined with other biomarkers, such as pathological forms of alpha-synuclein, as part of a biomarker panel^4^.

When disease state value of the biomarkers was assessed, a significant positive correlation was observed between motor severity scales (MSD-UPDRS III score and H&Y stage) and both NFL and GFAP plasma levels, and additionally between MSD-UPDRS III score and total-alpha synuclein plasma levels. In contrast to total alpha-synuclein and NFL, few studies have explored GFAP levels in the context of PD. To our knowledge, we are the first to report significant positive correlation between plasma GFAP levels and PD motor severity (MDS-UPDRS III scores and H&Y stage). Together these suggest that plasma GFAP levels may be a promising biomarker of PD progression now requiring further longitudinal validation. It has previously been suggested that astroglial GFAP is rapidly released into peripheral blood following axonal injury^7^ and, given the strong correlation between plasma NFL and GFAP levels in our study, our findings are likely to reflect this, although an increase due to enhanced GFAP gene expression cannot be ruled out. A previous study has shown that elevated CSF GFAP levels can differentiate PD patients progressing to cognitive decline during longitudinal follow up^20^, and more recently, a cross-sectional study has reported an association between serum GFAP levels and cognitive decline in PD patients^15^. The same study also reported significantly higher levels of serum GFAP in dementia with Lewy body patients compared to PD^15^, supporting the concept that GFAP levels may be associated with cognitive impairment in Lewy body disorders. Due to the cross-sectional nature and limited clinical data available in our study, however, we were unable to verify these findings in the present study.

As seen with GFAP, both NFL and total alpha-synuclein levels were associated with motor severity in our study, suggesting that both NFL and total alpha-synuclein levels may also be potential markers of PD progression. In line with our findings, total alpha-synuclein levels have been associated with higher MDS-UPDRS III score^36^ and shown to increase with longitudinal assessment in PD patients^26^. Further, higher NFL levels in a cross-sectional context have been associated with a significant increase in MDS-UPDRS motor scores in patients with postural instability and gait disorders (PIGD)^6^, whereas increasing serum NFL levels assessed in a large longitudinal multi-centre PD study were reported to be associated with increased MDS-UPDRS III scores^5^. We further reported a significant correlation between total-alpha synuclein and NFL levels, suggesting a relationship between the two biomarkers. This relationship has been highlighted in animal models of PD, where experimental induction of alpha-synuclein deposition in neurons has been associated with marked increases in CSF and blood derived NFL levels^37^. Elevated NFL CSF levels have further been reported in human studies, and associated with more severe PD motor symptoms^15^. With multiple reports additionally demonstrating that CSF and serum NFL levels are significantly correlated^4,5,34,37^, these findings are likely translatable to blood derived NFL levels.

In summary, this study assessed differences between serum and plasma levels of the neuronal damage markers NFL, GFAP, total tau and UCHL1. Limitations of the study were that it was a cross-sectional, single site study with a small sample size. An advantage of our study was the use of the SIMOA neurology 4-plex-A assay to assess all biomarkers in a common sample set and assay matrix, to ensure that cohort differences, pre-analytical variables and inter-assay variability did not influence results of the comparative study. Whilst no significant differences were observed between serum and plasma samples for NFL and GFAP, total tau levels were significantly higher and more stable in plasma samples. Thus, we determined that plasma samples were optimal to use when multiplexing the measured neuronal markers in a single assay run. Whilst none of the measured biomarkers were individually effective in discriminating PD patients from controls, a significant positive association between NFL, GFAP and total alpha-synuclein and PD motor severity was observed, suggesting that once the disease process is underway, they may be promising PD motor progression markers. Although their assessment in a larger longitudinal cohort will be required to clarify their translational use.

## Materials and methods

### Participant details and sample collection for plasma vs serum study

All methods were carried out in accordance with relevant guidelines and regulations. Participants were recruited with informed consent from the Parkinson’s Disease research clinic at the Brain and Mind Centre, University of Sydney. The study was conducted with ethical approval from the University of Sydney Human Research Ethics Committee (#2017/826). To compare different matrices, plasma and serum samples were simultaneously obtained from 12 subjects (6 healthy controls, 2 iRBD and 4 PD patients). Venous blood was collected in 10 mL EDTA tubes (plasma) (BD Biosciences) and 8 mL SST II Advance tubes (serum) (BD Biosciences) and centrifuged at 15,000 × g for 15 min at room temperature. Plasma and serum were then obtained from respective tubes, aliquoted and snap-frozen at 80 °C until use.

### Participant details and plasma samples collection for biomarker study

Participants were recruited with informed consent and the study was conducted with ethical approval from the University of Sydney Human Research Ethics Committee (#2016/363). The cohort included 29 PD cases which were diagnosed according to clinically established criteria^38^, which included motor assessments (MDS-UPDRS III scores and H&Y stage)). Additionally, the cohort included 30 age and sex matched control participants who had no neurological, psychiatric or immunological conditions, and with no first-degree relatives diagnosed with PD (demographic and clinical data shown in Table 1). To obtain plasma samples, venous blood was collected into 8 ml CPT vacutainers (BD Biosciences) in a non-fasted state and centrifuged at 1800 × g for 20 min at room temperature as previously described^39^. Plasma was then collected, snap-frozen into aliquots and stored at − 80 °C until used for assays at the completion of recruitment.

### Neurology 4-Plex-A SIMOA assay

The ultrasensitive single molecule array (SIMOA) Human Neurology 4-Plex A assay (Quanterix; Cat. No. 102153) and Quanterix HD-1 Analyzer (Quanterix) were used for the assessment of levels of NFL, tau, GFAP and UCHL1. Briefly, sample aliquots were sent to GeneWorks (originally Adelaide, SA; relocated to Melbourne, VIC) on dry ice with the Quanterix assay performed on a fee for service basis following the manufacturer instructions and a 1:4 dilution of sample. A 7-point calibration curve and quality controls for each analyte were included. The calibration curve range for each analyte were as follows; 0–450 pg/mL (NFL), 0–9000 pg/mL (GFAP and UCHL1) and 0–90 pg/mL (Tau). Concentrations of the respective analytes in biosamples and quality controls were interpolated from the calibration curve using a cubic curve fit (1/Y^2^ weighted). Duplicates were used for intra-assay and inter-assay precision testing. A CV > 12% cut-off was chosen based on the assay datasheet which reported a maximum within assay CV of 11.3% (serum panel tested for UCHL1). Additionally, past studies which indicate an intra-assay CV between 10 to 13.7% as good precision^40–44^. We therefore took 12% to be a suitable cut-off.

### Assessment of plasma total alpha-synuclein and hemoglobin

Total plasma alpha-synuclein levels were assessed using the U-PLEX Plus Human alpha-synuclein kit (Mesoscale Discovery, Cat. No. K151WKP-2) as previously described^21^ using 1:8 dilution of the plasma samples. An 8-point calibration curve and 3 quality controls provided in the kit were included in each assay plate. The calibrator curve range was 0–10,500 pg/mL and was used to interpolate concentrations of total alpha-synuclein from plasma samples and quality controls. As plasma hemoglobin (Hgb) levels are important to include as a covariate in the analysis of total alpha synuclein in plasma samples^21^, Hgb levels were also measured using a Human Hemoglobin ELISA Kit (Abcam; cat# ab157707) at a 1:1000 dilution of plasma, as per the manufacturer instructions. Hgb concentrations were interpolated from a 6-point calibration curve ranging from 0 to 200 ng/mL using a 4-parameter logistic curve fit. Inter-assay CV% for total alpha-synuclein and Hgb were < 15% and < 10% respectively.

### Statistical analysis

All statistical analyses were performed using the IBM SPSS Statistics 26 software with significance determined at a *p* level < 0.05. Corrections for multiple comparisons were not performed. All plots were generated using GraphPad Prism v 8.4.3. A Mann Whitney U test was used to assess differences between demographic and clinical data. Multivariate analysis covarying for age, sex and Hgb levels was also employed to assess differences between total alpha-synuclein in PD and controls, as previously suggested^21^. Mann Whitney U test was used to assess differences between serum and plasma levels of tau, GFAP, NFL and UCHL1, and Box-plot analysis was used to assess outliers, which were defined as values less than Q1- (5 × IQR) or greater than Q3 + (5 × IQR). A total of 1.7% of the NFL data set, 3.4% of the tau data set and 6.8% of the UCHL-1 data set were defined as outliers and values replaced with the mean for that group. No outliers were identified for GFAP. For NFL and GFAP. Concentration values were log transformed to achieve a normal distribution as assessed by Shapiro–Wilk test (*p *> 0.05). A parametric t-test was then used to assess differences between control and PD plasma concentrations for GFAP, NFL and tau. As UCHL1 could not be transformed to achieve normality, Mann Whitney U was used to assess differences between control and PD plasma concentrations. Multivariate analysis covarying for age and sex was used to determine any differences between biomarker levels of NFL, GFAP and tau in controls and PD patients. Hemoglobin levels were included as a covariate in multivariate analyses employing alpha-synuclein. Spearman correlation analyses were performed to determine associations between biomarkers, clinical and demographic data. Partial correlation analysis using hemoglobin as a covariate was used for any correlation involving alpha-synuclein.

## Supplementary Information

Supplementary Information.

## Data Availability

All data generated or analysed during this study are included in this published article (and its Supplementary Information files).
