# Supplementary material for: Evaluation of plasma levels of NFL, GFAP, UCHL1 and tau as Parkinson's disease biomarkers using multiplexed single molecule counting
Source: Sci Rep. 2023 Mar 30;13:5217. doi: 10.1038/s41598-023-32480-0 (PMC10063670; doi:10.1038/s41598-023-32480-0)
Supplement: Supplementary file 1 — Supplementary Information. [file 41598_2023_32480_MOESM1_ESM.pdf]

Evaluation of plasma levels of NFL, GFAP, UCHL1 and tau as Parkinson's disease biomarkers using multiplexed single molecule counting

Priscilla Youssef[a], Laura Hughes[a], Woojin S. Kim[a], Glenda M. Halliday[a], Simon J. G. Lewis[a], Antony Cooper[b], Nicolas Dzamko[a]*

a) School of Medical Sciences, Faculty of Medicine and Health and the Brain and Mind Centre, University of Sydney, Camperdown, NSW, 2050, Australia.
b) Garvan Institute of Medical Research.

Running title: Plasma neurodegeneration markers in Parkinson's disease.

***Correspondence to**: Nicolas Dzamko, nicolas.dzamko@sydney.edu.au, School of Medical Sciences, University of Sydney, Camperdown, NSW, 2050, Australia.

Keywords: Parkinson's disease, SIMOA, biomarker, plasma, neurofilament

Supplementary data

Supplementary Figure 1

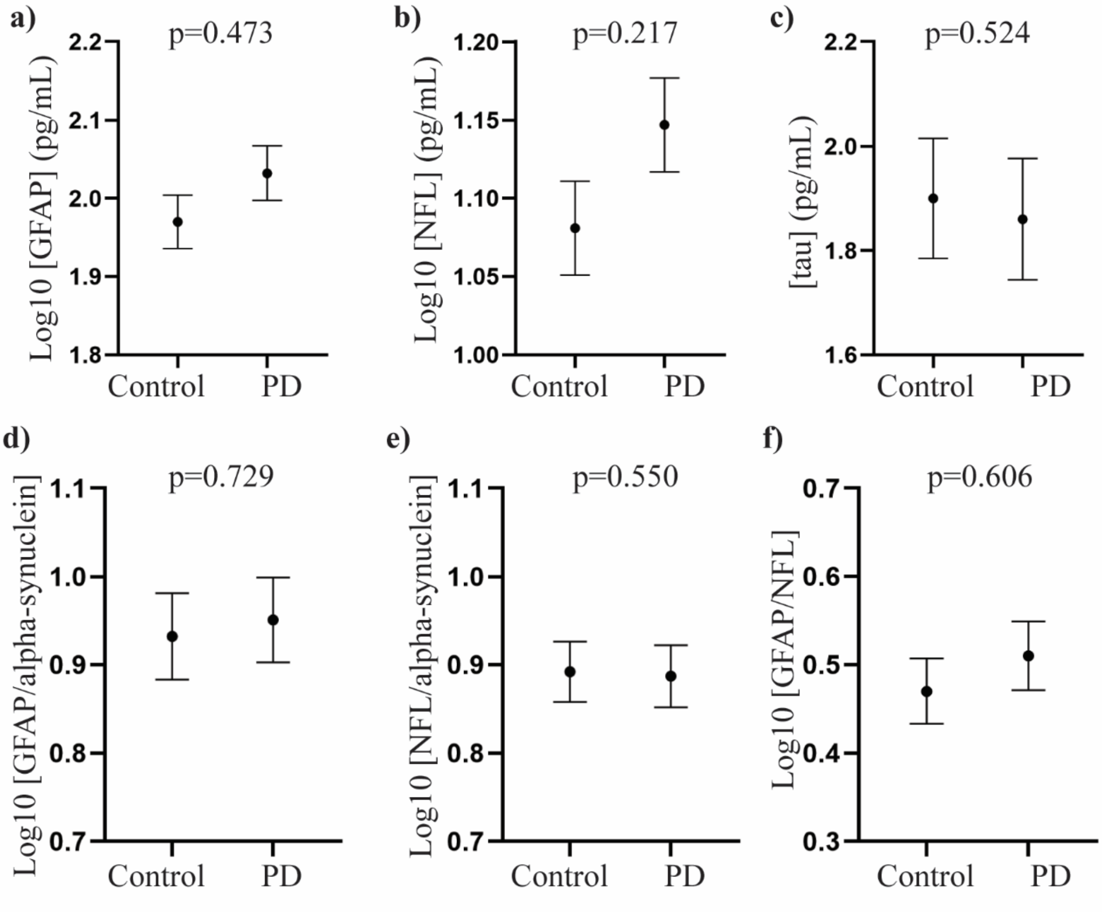

Supplementary Figure S1. Covaried analysis of GFAP, NFL, and tau, and the ratio of GFAP, NFL and alpha-synuclein in control and PD plasma samples. Where required, data were transformed to achieve normality. Multivariate analysis revealed no significant difference between control and PD patient plasma levels of (a) GFAP, (b) NFL and (c) tau, after covarying for age and sex. When exploring differences between biomarker ratios, multi-variate analysis revealed no significant difference between the ratio of (d) GFAP and alpha-synuclein, and the ratio of (e) NFL and alpha-synuclein, after covarying for age, sex and hgb levels. Univariate analysis also revealed no significant difference between the ration of (f) NFL and GFAP, after covarying for age and sex. Post-analysis graphs display the estimated marginal mean ± SE (n=30 controls, n=29 PD).
